# Graphite Felt Decorated with Metal–Organic Framework-Derived Nanocomposite as Cathode for Vanadium Redox Flow Battery

**DOI:** 10.3390/nano15070535

**Published:** 2025-04-01

**Authors:** Priya Lakshmanan, Chia-Hung Huang, Suba Devi Rengapillai, Yong-Song Chen, Wei-Ren Liu, Cheng-Liang Hsu, Sivakumar Marimuthu

**Affiliations:** 1 #120, Energy Materials Lab, Department of Physics, Science Block, Alagappa University, Karaikudi 630003, Tamil Nadu, India; priyalj1998@gmail.com; 2 Metal Industries Research and Development Centre, Kaohsiung 81160, Taiwan; chiahung@mail.mirdc.org.tw (C.-H.H.); clhsu@mail.nutu.edu.tw (C.-L.H.); 3 Department of Mechanical Engineering and Advanced Institute of Manufacturing with High-Tech Innovations, National Chung Cheng University, Chiayi 621301, Taiwan; imeysc@ccu.edu.tw; 4 Department of Chemical Engineering, R&D Center for Membrane Technology, Chung Yuan Christian University, 200 Chung Pei Road, Taoyuan 32023, Taiwan; wrliu@cycu.edu.tw; 5 Hierarchical Green-Energy Materials (Hi-GEM) Research Center, National Cheng Kung University, 1 University Road, Tainan 70101, Taiwan

**Keywords:** electrocatalytic efficiency, energy efficiency, electrode modification, graphite felt, polarization loss

## Abstract

Fabricating electrodes with high electrocatalytic efficiency is crucial for the commercial feasibility of vanadium redox flow batteries (VRFBs). In this study, metal–organic framework-derived ZnO and Fe_2_O_3_ with a high specific surface area were successfully synthesized via high-energy ball milling. The nanocomposite material (ZnO-Fe_2_O_3_) was prepared through ultrasonication and coated on the graphite felt using dip coating, serving as the positive electrode for the VRFB. These modified electrodes control polarization losses, leading to high voltage efficiency (VE) and energy efficiency (EE), even at high current densities. Consequently, the nanocomposite-modified electrode shows VE of 87% and EE of 84% at 50 mA/cm^2^, surpassing the performance of individual materials. The nanocomposite material retains its EE without degradation over 250 cycles at a current density of 150 mA/cm^2^. This enhanced performance is due to improved kinetics and reduced losses in the VO^2+^/VO_2_^+^ redox couple, enabled by the nanocomposite material.

## 1. Introduction

The growing demand for electricity projected for the coming decades has heightened the interest in developing new technologies for energy production from renewable sources, such as wind and solar power. For these renewable energy sources to be successfully integrated into the grid, there must be competitive energy storage solutions for load leveling and peak shaving. This integration can address the challenge of the unpredictable and intermittent nature of renewable energy production [1]. Electrochemical devices, such as batteries and supercapacitors, have demonstrated higher efficiencies for electrical energy storage. One technology particularly emerging as a promising technology is the redox flow battery (RFB) for static energy storage systems due to its high capacity, scalability, durability, and ease of maintenance [2]. Unlike conventional batteries, which store energy in a confined case, RFBs exhibit electroactive materials (electrolytes) in external chambers. These electrolytes are pumped to the electrode surfaces, where redox reactions occur, making the system ideal for large-scale energy stowage applications. Increasing the volume and concentration of the external chambers can significantly enhance energy capacity, and the number of the electrons transferred during the redox reactions can further improve overall performance [3]. Among the different types of RFBs, vanadium redox flow batteries (VRFBs) have become particularly noteworthy due to their high stability and minimized risk of irreversible cross-contamination because the same vanadium species is present in both anolyte and catholyte. However, although Fe-based RFBs are even available at low-cost, they suffer from irreversible crossover and degradation. Hence, VRFBs offer better efficiency (~80–85%) and superior reaction kinetics, making VRFBs a leading commercial RFB technology. However, vanadium ion crossover through Nafion membrane remains a challenge, leading to capacity fading [4]. Efforts to improve membrane selectivity, electrolyte rebalancing, and system optimization help to mitigate these issues. Moreover, VRFB technology has been extensively tested in commercial applications, leading to industrial optimizations and widespread deployment. These factors collectively make VRFBs the most commercially viable RFB technology today.

VRFBs use the same vanadium species in both the anolyte and catholyte, which prevents the cross-contamination of electroactive materials. Furthermore, vanadium can be recovered from industrial waste, such as mine tailings and ash, providing an environmental benefit. Recent literature highlights the achievement of Uni Energy Technologies (UET) in creating the world’s largest battery and largest VRFB storage facility, with a capacity of 200 MW and 800 MWh, respectively, located in North China [5].

For commercial applications, researchers are focused on enhancing the power capacity and improving the energy efficiency (EE) of redox flow batteries (RFBs). Several factors contribute to the increased power density of RFBs, including a larger electrode surface area, voltage efficiency, and the size and number of electrode cells or modules [6]. Consequently, the synthesis of suitable electrodes with high electronic conductivity, which minimizes ohmic drop, along with an enhanced surface area and hydrophilicity for vanadium redox flow battery (VRFB) reactions, is a primary focus for researchers. In recent times, graphite felts (GFs) [7] and carbon felts (CFs) [8] have been utilized as electrode materials due to their superior electronic conductivity and stability. However, these materials have limited redox activity, specifically towards the VO^2+^/VO_2_^+^ redox couple at the positive electrode and the V^2+^/V^3+^ redox couple at the negative electrode. Additionally, they suffer from low surface area, VE, and overall EE. To address these challenges, various metals and metal oxides (such as Ir, [9] Bi, [10] Sn, [11] Cu, [12], and Cr_2_O_3_ [13]) have been incorporated into GFs as electrode for VRFBs. However, these modifications often result in reduced electronic conductivity. In this work, GF was chosen over CF as the electrode material due to its superior electrochemical properties, such as high electrical conductivity due to its more ordered graphitic structure, which facilitates efficient electron transport and reduces charge transfer resistance. Additionally, GF demonstrates strong oxidation and corrosion resistance, making it more stable in the highly oxidative VRFB environment and ensuring long-term durability. Furthermore, GF exhibits high intrinsic catalytic activity compared to CF, further improving reaction kinetics and overall battery efficiency. However, the large-scale application of GF is limited by its poor hydrophilicity and electrochemical activity [14]. Therefore, we subjected it to thermal treatment, followed by nanocomposite modification using dip coating. These combined advantages make GF a more suitable electrode substrate for achieving superior electrochemical performance in VRFBs.

In this study, we present a novel approach to synthesizing a composite material composed of MOF-derived ZnO and Fe_2_O_3_ using high-energy ball milling followed by ultrasonication. The nanocomposite, subsequently coated onto GF surfaces via dip coating, exhibits exceptional stability as an electrocatalyst. Compared to conventional pure metal oxides such as ZrO_2_, [15] PbO_2_, [16], and Ta_2_O_5_ [17], MOF-derived metal oxides offer a significantly enhanced specific surface area and long-term durability. As we stated in the Results and Discussion sections, the individual MOF-derived metal oxides exhibit comparatively low electrochemical activity when compared to nanocomposite materials. This result reveals that the ZnO-Fe_2_O_3_ nanocomposite/GF system demonstrates robust electrocatalytic activity towards the VO^2+^/VO_2_^+^ and V^2+^/V^3+^ redox couples, highlighting its potential for applications in energy storage systems. This work underscores the superior performance of MOF-derived nanocomposite metal oxides and emphasizes the imperative for further research and development in this promising field.

## 2. Materials and Methods

### 2.1. Materials

Zinc acetate dehydrate (Zn(O_2_CCH_3_)_2_(H_2_O)_2_), iron acetate (Fe(CO_2_CH_3_)_2_), terephthalic acid (C_8_H_6_O_4_), dimethylformamide (DMF, (CH_3_)_2_NCH), and ethanol (C_2_H_5_OH) were purchased from Sigma Aldrich for synthesis, without any additional purification.

### 2.2. Preparation of Zn-MOF Derived ZnO

Zn-MOF-derived ZnO was prepared using a high-energy ball milling reaction. Stochiometric amounts of zinc acetate dehydrate (4.3 g, 20 mmol), terephthalic acid (1.6 g, 10 mmol), zirconium balls (118 g), and DMF (5 mL) were transferred into a ball milling tank. Then, the constant rotation of 1 h was maintained, and a 10 min relaxation period was applied during milling at 245 rpm. Then, the slurry was washed with DMF and kept in the oven at 90 °C overnight. After drying, we obtained the Zn-MOF precursor. At that time, we kept the precursor for calcination at 600 °C for 2 h to obtain ZnO.

### 2.3. Preparation of Fe-MOF-Derived Fe_2_O_3_

Molar amounts of iron acetate (2.3 g, 20 mmol) and terephthalic acid (1.6 g, 10 mmol) were subjected to milling at 250 rpm for 30 min. The synthesis process of Fe-MOF followed the same steps as Zn-MOF. Then, the precursor was calcined at 800 °C for 4 h to attain Fe_2_O_3_.

### 2.4. Preparation of ZnO-Fe_2_O_3_ Nanocomposite

The synthesized ZnO and Fe_2_O_3_ were used as precursors for nanocomposite preparation. Initially, 0.5 g of ZnO and 0.5 g of Fe_2_O_3_ were distributed in deionized water by stirring. Consequently, the prepared homogeneous solution was exposed to ultrasonication for 2 h to obtain a nanocomposite. Finally, the solution was dried in an oven at 80 °C for 12 h to obtain a pure material.

### 2.5. Preparation of ZnO-Fe_2_O_3_ Nanocomposite Modified GF

**Figure 1:**
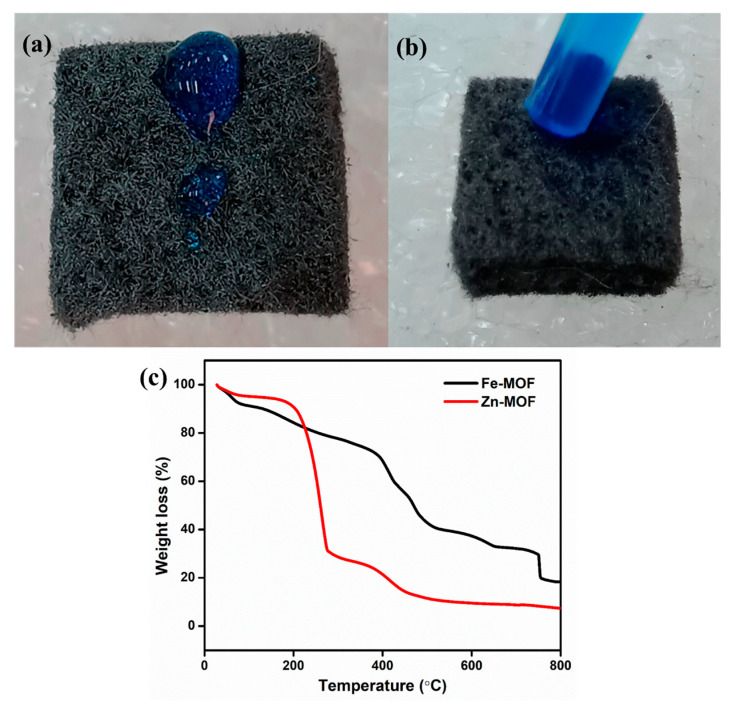
(**a**) Pristine GF (hydrophobic), (**b**) coated GF (hydrophilic), (**c**) TGA of the prepared precursor.

Pristine GF (6.5 mm thickness, (GF065) obtained from CeTech, Taipei, Taiwan) was used for the synthesis process. The common tactic used for GF, which is thermal activation under air, improves the surface roughness, while the hydrophobic in nature is the main issue for pristine GF. Therefore, the GF endured a thermal action at 500 °C for 6 h within a tubular furnace. Subsequently, the GF was loaded using the dip coating method. Initially, the prepared ZnO, Fe_2_O_3_, and ZnO-Fe_2_O_3_ solutions were prepared by mixing polyvinylidene fluoride (PVdF) (20%), and carbon black (20%) in N-methyl pyrrolidone (NMP) solvent. For a better dissolvement and clear solution, we performed probe sonication for 3 h. Then, the thermally treated GF was immersed into the separate solution, with a mass loading of 3 mg/cm^2^. The loaded GF was set aside in an oven at 80 °C overnight. Then, the electrolyte was dropped onto pristine GF, which exhibited hydrophobic behavior, as shown in Figure 1a. After the electrode was coated onto the GF, it became highly hydrophilic; that is, the electrolyte was absorbed immediately, as clearly seen in the digital image (Figure 1b).

### 2.6. Characterizations Techniques

The thermal stability of the prepared precursors was assessed using Thermogravimetric Analysis (TGA) with a Perkin Elmer diamond TG-DTA instrument, Karaikudi, India. The crystallinity phases of the as-prepared materials were identified through X-ray diffraction (XRD) utilizing a D8 discover instrument (Karaikudi, India) equipped with a GADDS system. The morphological images were analyzed using field emission scanning electron microscopy (FESEM, EV018, CARL ZEISS, Krishnarkoil, India), high-resolution transmission electron microscopy (HRTEM, Themis-Z 3.1, TFS, USA, Chiayi, Taiwan), and JEM 2010, Jeol (Tokyo, Japan). Nitrogen adsorption–desorption isotherms (Brunauer–Emmett–Teller (BET) of the prepared materials were measured at 77 K using an ASAP 2020 adsorption analyzer (Micromeritics, Bangalore, India).

Electrochemical characterizations, such as cyclic voltammetry (CV) and electrochemical impedance spectroscopy (EIS) were performed in the frequency range of 10 kHz–10 mHz using a three-electrode system consisting of a platinum counter electrode, an Ag/AgCl reference electrode, and the prepared material as the working electrode, with a Zennium ZAHNER (Chiayi, Taiwan) workstation. Both three-electrode and flow cell tests were performed using a solution of 1.6 M VOSO_4_ and 4.3 M H_2_SO_4_, which was purged with nitrogen gas prior to use in electrochemical applications. The flow cell test was conducted with catalyst-modified electrodes with a surface area of 25 cm^2^. To assess persistence, a vanadium redox flow battery (VRFB) test was conducted for both individual and nanocomposite materials. Nafion 212 (proton exchange membrane) was used as a separator to eliminate the cross-contamination between the electrode and the electrolyte. The electrolytes were maintained at a constant flow rate of 1.5 L/h at 100 rpm using a dual-channel peristatic pump. The concentration and volume (50 mL) of both the anolyte and the catholyte were upheld throughout the charging/discharging process. Flow cell performance was evaluated using a battery tester (PFX 2021, Kikusui, Japan). The current density was varied from 100 mA/cm^2^ to 250 mA/cm^2^ sustained at a constant voltage of 0.7–1.69 V.

## 3. Results and Discussion

To obtain metal oxides derived from MOFs, the thermal stability of Zn-MOF and Fe-MOF was optimized using TGA under nitrogen at a heating rate of 1 °C/min. Figure 1c shows the TGA curves for both materials. For Zn-MOF, two weight loss events occurred with increasing temperature. The first weight loss of about 60% below 210 °C can be attributed to the removal of moisture in the sample. The second weight loss, between 210 °C and 420 °C, is approximately 17% and is mainly due to the structural decomposition of the Zn-MOF. Beyond 450 °C, no further weight loss was observed, indicating the completion of the calcination process, as optimized by TGA. Similarly, Fe-MOF exhibits three distinct weight loss stages. The first weight loss of 9% occurred below 170 °C, due to the evaporation of the water content in the sample [18]. The second weight loss, which occurred between 170 °C and 410 °C, was 32% and is attributed to the decomposition of the organic ligand (BDC). As the temperature increased further, the third weight loss, occurring between 410 °C and 720 °C, can be ascribed to the loss of acetate, structural decomposition, and the conversion of Fe-MOF into FeO_x_ [19]. After 750 °C, the weight loss was stable. Therefore, the calcined product of Zn-MOF at 500 °C and Fe-MOF at 800 °C was used as an effective material for energy storage application.

**Figure 2:**
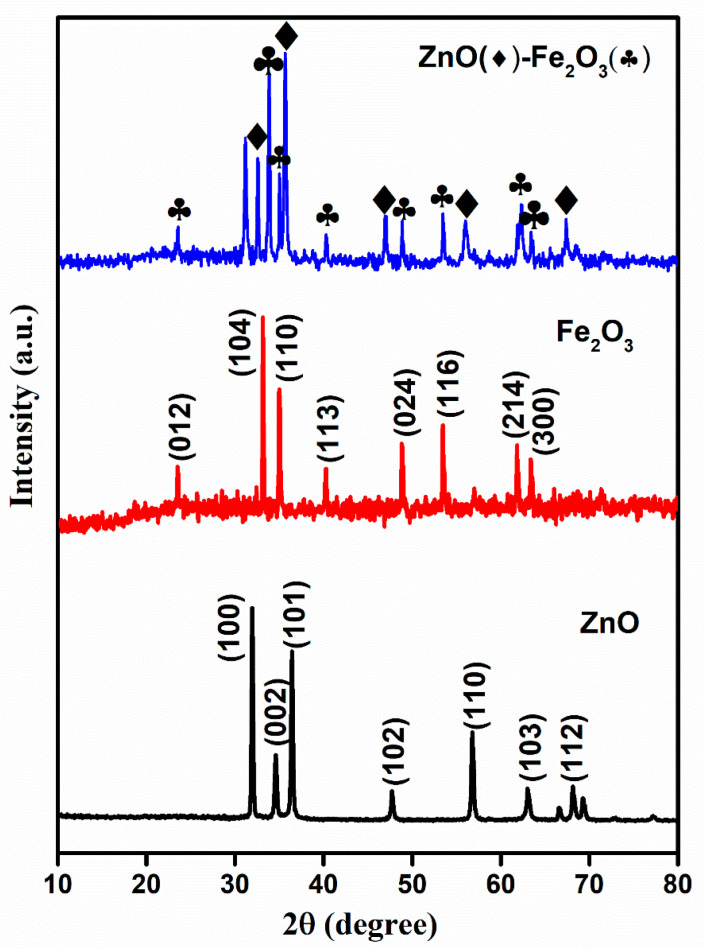
XRD spectra for ZnO, Fe_2_O_3_, and ZnO-Fe_2_O_3_ nanocomposite.

The crystallinity and phase structure of the calcined precursor materials were analyzed using X-ray diffraction (XRD). The MOF-derived ZnO, Fe_2_O_3_, and ZnO-Fe_2_O_3_ nanocomposite patterns are illustrated in Figure 2. The diffraction peaks of ZnO correspond to the JCPDS: 36-1415, which is indicative of a well-ordered hexagonal wurtzite structure with lattice constants a = 3.25 Å and c = 5.21 Å. The principal peaks located at 2θ = 31.7, 34.3, 36.2, 47.5, 56.5, 62.8, and 67.9° correspond to the crystallographic planes (100), (002), (101), (102), (110), (103), and (112), respectively, which are consistent with ZnO (Figure 2) [20]. The Fe-MOF-derived rhombohedral Fe_2_O_3_ phase exhibited characteristic peaks indexed to JCPDS: 24-0072, with lattice constants a = 5.11 Å and c = 13.82 Å, indicating a lower degree of crystallinity, as depicted in Figure 2. The observed peaks at 2θ = 23.6, 32.9, 34.9, 40.1, 48.7, 53.5, 61.8, and 62.9° correspond to the (012), (104), (110), (113), (024), (116), (214), and (300) planes, respectively (Figure 2) [21].

ZnO-Fe_2_O_3_ nanocomposite upheld a crystallographic profile that is a combination of ZnO and Fe_2_O_3_, as confirmed by the sharp diffraction peaks at 36.2° (ZnO), marked by diamonds, and 32° (Fe_2_O_3_), marked by clubs (Figure 2). The peaks attributed to Fe_2_O_3_ minimized the intensity in the nanocomposite, suggesting that hematite was dispersed within the ZnO matrix. The obtained results align well with the parameters reported in previous literature [22]. However, the individual materials were calcined at appropriate temperatures, indicating the decomposition of the MOF structure into metal oxides, with carbon encapsulating into the metal oxides. In general, the presence of amorphous or secondary phases in the material results in resistive interfaces, which lower the efficiency. In the present case, the use of two different metal oxide species enhances the synergistic effects of the two metal oxide species, fostering deeper electrochemical activity and improved redox kinetics compared to the single-species system. The obtained higher crystallinity and phase composition of the material influence its conductivity and improve its voltage efficiency and electrochemical stability during charge/discharge [23].

**Figure 3:**
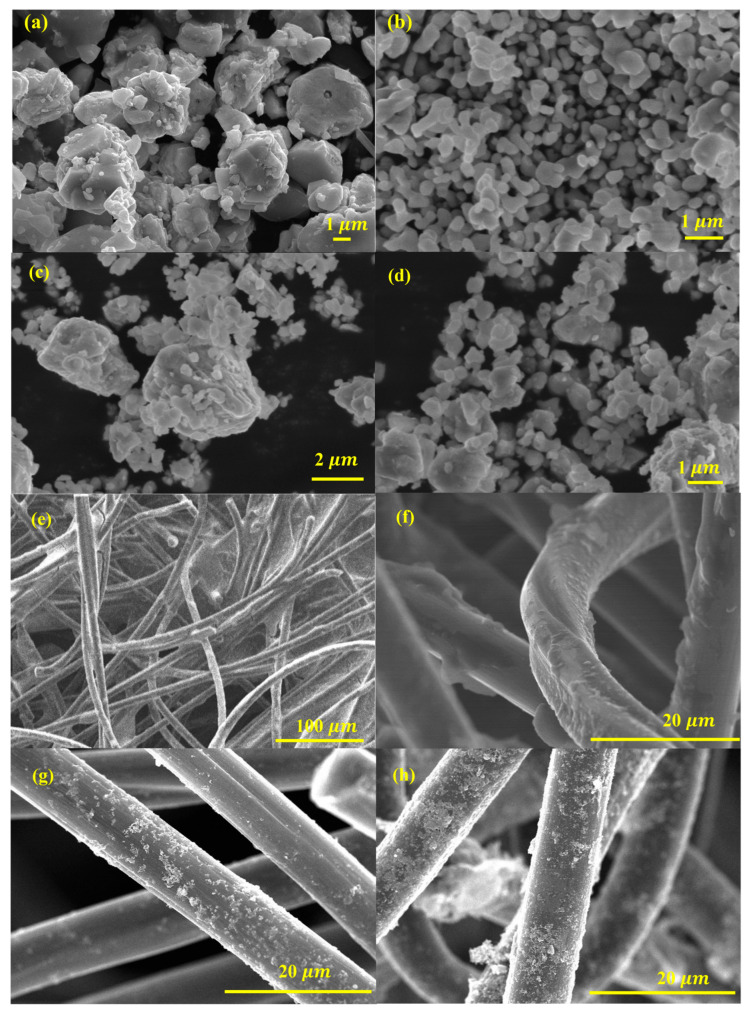
FE-SEM images (**a**) ZnO, (**b**) Fe_2_O_3_, (**c**,**d**) ZnO-Fe_2_O_3_ nanocomposite, (**e**) HGF, (**f**) ZnO-loaded HGF, (**g**) Fe_2_O_3_-loaded HGF, and (**h**) ZnO-Fe_2_O_3_-loaded GF.

The FE-SEM images provide critical insights into the morphology and surface characteristics of ZnO, Fe_2_O_3_, and ZnO-Fe_2_O_3_ nanocomposite derived from MOF precursors. Figure 3a illustrates ZnO particles, revealing a well-defined, hexagonal morphology with a textured surface. These ZnO particles exhibit uniform size distribution, with lengths ranging from a few hundred nanometers to several micrometers and diameters in the range of 5–10 µm. The surfaces of the particles are smooth, indicating a high degree of crystallinity. The hexagonal morphology of ZnO is beneficial for applications in catalysis and energy storage due to the high surface area and effective charge transport properties. Figure 3b depicts Fe_2_O_3_, revealing that the particles are predominantly spherical, with some degree of agglomeration. The Fe_2_O_3_ particles have diameters ranging from 1 to 2 µm. The surface texture of these particles appears rough, which could be indicative of the polycrystalline nature of Fe_2_O_3_. The spherical morphology is advantageous for enhancing electrochemical activity due to the large surface area available for reactions [24]. Figure 3c,d display the ZnO-Fe_2_O_3_ nanocomposite, where a combination of spherical structures and hexagonal particles, highlights the successful integration of ZnO and Fe_2_O_3_ phases. The nanocomposite particles ZnO and Fe_2_O_3_ are interconnected, forming a network-like structure. This hybrid morphology is advantageous for VRFB applications, as it combines the high surface area of Fe_2_O_3_ with the excellent charge transport properties of ZnO [25]. The well-distributed nanostructure can improve vanadium ion adsorption, enhance charge transfer, and reduce polarization losses in VRFBs.

The surface morphology of the GF decorated with the synthesized material is illustrated in Figure 3. The synthesized material forms a uniform coating on the GF surface, indicating its potential for consistent electrochemical performance. Figure 3e shows the fibrous nature of the felt, with individual fibers intertwined to form a 3D network. The heat treatment likely enhances the electrical conductivity and stability of the felt, making it a suitable scaffold for loading with metal oxides. When loaded with ZnO (Figure 3f), the thermally treated GF (HGF) exhibits ZnO uniformly dispersed on the surface of the graphite felt. The ZnO appears as small particles that are well-integrated with the graphite felt. This uniform distribution ensures effective contact between the ZnO and the electrolyte in electrochemical applications, facilitating efficient electron transport and catalytic activity. In Fe_2_O_3_-loaded HGF (Figure 3g), the FE-SEM image reveals spherical Fe_2_O_3_ nanoparticles attached to the graphite felt. These nanoparticles have a relatively uniform size and are evenly distributed across the felt. The well-distributed metal oxide particles across the GF surface are vital for maximizing the electrocatalytic active sites available for redox reactions. The nanocomposite-loaded HGF (Figure 3h) shows a combination of ZnO and Fe_2_O_3_ nanoparticles dispersed on the graphite felt. The FE-SEM image highlights the synergistic integration of ZnO and Fe_2_O_3_, forming a hybrid structure that combines the advantages of both materials. The nanocomposite material displays a smooth surface, in stark contrast to the rough and uneven surfaces observed in the GF and the individually coated metal oxide material. The rough and uneven surface of the GF highlights the presence of surface defects and irregularities, which are significantly reduced in the nanocomposite material (Figure 3h). The smooth surface of the nanocomposite material suggests enhanced interaction and adhesion between the GF and the coated material [26]. The ZnO-Fe_2_O_3_ nanocomposite acts as a template, facilitating the formation of a smooth surface, while also contributing to the electrocatalytic activity in the VRFB. This modification increases the specific surface area, enhances electrolyte penetration, and introduces more electroactive sites, improving overall energy efficiency. The combination of ZnO-Fe_2_O_3_ on the conductive substrate enhances the material’s electrochemical properties, making it a promising electrode for VRFBs. The modified electrode structure provides more active sites for vanadium ion interactions, leading to lower polarization losses and improved reaction reversibility. Consequently, these morphological improvements lead to higher voltage efficiency (VE) and energy efficiency (EE), making ZnO-Fe_2_O_3_-modified GF ahighly effective electrode material for VRFB applications [27].

**Figure 4:**
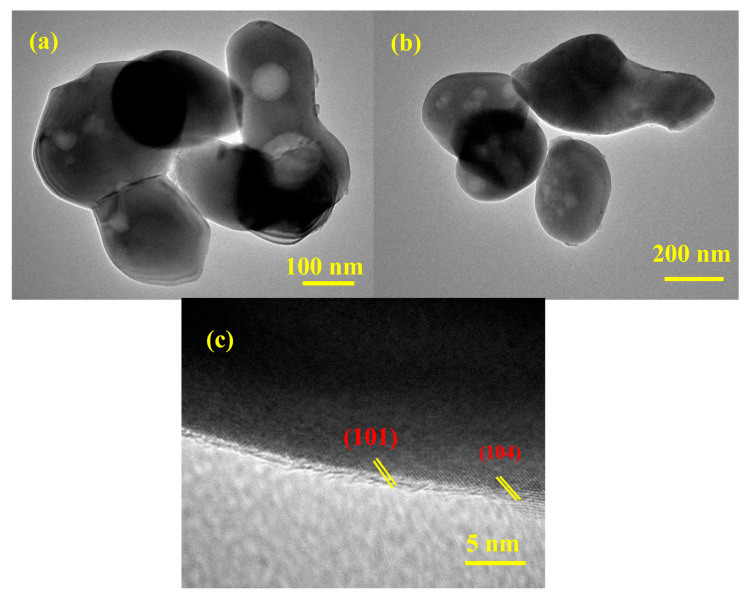
(**a**–**c**) HR-TEM images of the ZnO-Fe_2_O_3_ nanocomposite.

The HR-TEM analysis provides critical insights into the morphology, nanostructure, and crystalline nature of the as-prepared material. The HR-TEM images of the synthesized ZnO-Fe_2_O_3_ nanocomposite derived from MOF routes for VRFBs (Figure 4) reveal irregular, agglomerated particles ranging from 100 to 200 nm. The material exhibits a high degree of polydispersity among the particles, indicating that the synthesis process might result in the formation of particles with varying sizes. The bright-field images (Figure 4a,b) show contrast variations, including black dots, indicating density or compositional differences within the nanoparticles. These black dots are likely regions of higher electron density, suggesting the presence of ZnO and Fe_2_O_3_ nanoparticles within the composite matrix [28]. The high-resolution image (Figure 4c) confirms the crystalline nature of the material, with well-defined lattice fringes, showing d-spacing values of approximately 0.24 and 0.26 nm, corresponding to the (104) and (110) planes in the XRD. These findings emphasize the successful transformation of the MOF precursor into a crystalline ZnO-Fe_2_O_3_ nanocomposite. The agglomerated morphology, while reducing the surface area, may enhance stability during electrochemical cycling [29]. The high crystallinity and specific crystallographic planes observed are critical for electrocatalytic applications, providing active sites for electrochemical reactions. These observations highlight the potential of the ZnO-Fe_2_O_3_ nanocomposite for advanced catalytic and energy stowage applications, warranting further investigation into its performance under operational conditions.

**Figure 5:**
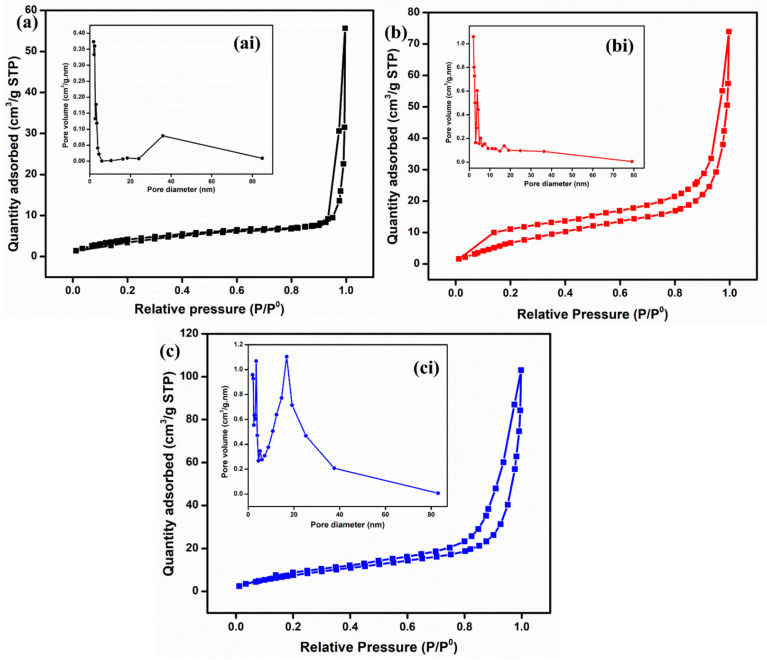
BET isotherm and pore distribution: (**a**) ZnO, (**b**) Fe_2_O_3_, and (**c**) ZnO-Fe_2_O_3_ nanocomposite.

To analyze the textural properties, such as specific surface area (SSA), pore volume, and pore size, BET analysis was conducted for all the materials, as shown in Figure 5. The specific surface areas calculated for ZnO, Fe_2_O_3_, and ZnO-Fe_2_O_3_ nanocomposite are 9.6, 13.3, and 23.2 m^2^/g, respectively. Based on the IUPAC classification, the adsorption–desorption isotherm indicates a type-IV with an H4-hysteries loop for all three samples [30]. Moreover, the pore diameters for all the materials, found to be between 10 and 16 nm, were determined using the Barrett–Joyner–Halenda (BJH) isotherm (inset of Figure 5ai,bi,ci). This indicates that all the materials are mesoporous in nature, which enhances ion diffusion and mitigates mass transport limitations. All the parameters are summarized in Table 1. Notably, the nanocomposite exhibits a higher specific surface area and porosity compared to the individual materials, which enhances ion migration due to the increased availability of active sites. The surface properties of Fe_2_O_3_, including its semiconducting nature and high surface area, can enhance the electrochemical activity for the nanocomposite electrode. This property is attributed to the inverse relationship between crystallinity and specific surface area, as well as the enhanced SSA observed in MOF-derived metal oxides [31]. The synergistic interaction between Fe_2_O_3_ and ZnO, coupled with a high surface area, significantly improves the kinetics of vanadium redox reactions. These factors collectively reduce the overpotential, leading to high voltage efficiency. In addition, it improves ion accessibility, charge transfer dynamics, and energy efficiency [32].

**Figure 6:**
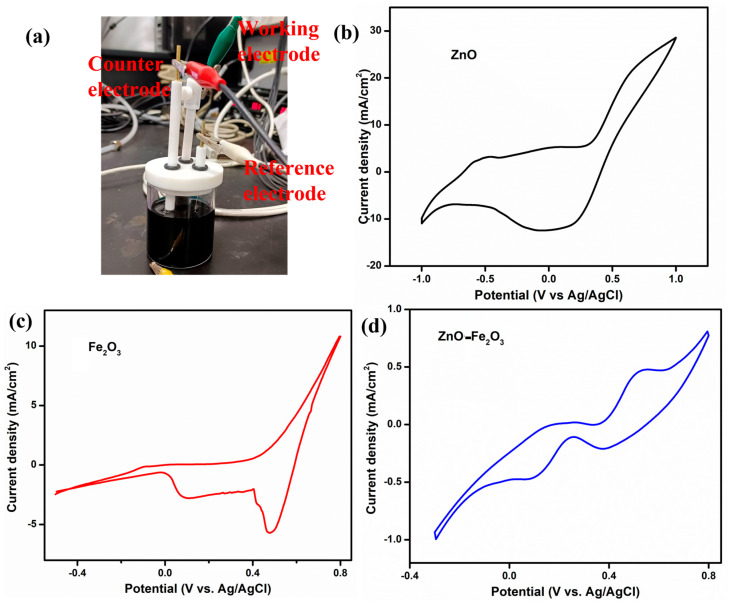
(**a**) Three-electrode system, CV curve, (**b**) ZnO, (**c**) Fe_2_O_3_, (**d**) ZnO-Fe_2_O_3_ nanocomposite.

To elucidate the role of the vanadium electrolyte and the as-prepared electrodes as working electrodes, CV was conducted using a three-electrode setup at a scan rate of 5 mV/s, with Pt as the counter electrode and Ag/AgCl as the reference electrode (Figure 6a). The CV profiles for ZnO, Fe_2_O_3_, and ZnO-Fe_2_O_3_ nanocomposite in a 1.6 M VOSO₄ and 4.3 M H_2_SO₄ electrolyte solution are depicted in Figure 6a–c. In Figure 6b, which illustrates the ZnO-modified GF electrode in the potential window of −1 V to +1 V, the anodic peak (oxidation) is observed at −0.49 V, while the cathodic peak (reduction) appears at 0.05 V. These peaks are indicative of the redox reactions involving V(IV) (VO^2+^) and V(V) (VO_2_^+^), respectively [33]. The pronounced separation between the anodic and cathodic peaks (ΔE_p_ = 0.54 V) suggests substantial overpotentials and sluggish electron transfer kinetics, implying that the redox process is not highly reversible (quasi-reversible) for this individual material. Figure 6c illustrates the CV profile for the Fe_2_O_3_-modified GF in the potential window of −0.4 V to +0.4 V, showcasing an anodic peak at −0.08 V and two reduction peaks at 0.47 and 0.1 V. The anodic peak corresponds to the oxidation of V(IV) (VO^2+^) to V(V) (VO_2_^+^). The relatively low (slightly negative) potential signifies that the Fe_2_O_3_ modified electrode more efficiently facilitates this reduction process by providing catalytic sites that diminish the overpotential required for the redox reaction compared to the ZnO modified electrode [34]. The cathodic peak at 0.47 V is attributed to the reduction of V(V) back to V(IV), while the second reduction peak at 0.1 V suggests an additional reduction step, likely the reduction of V(III) to V(II). The presence of two distinct reduction peaks indicates the occurrence of multiple redox processes. The considerable separation between the oxidation peak and the first reduction peak (ΔEp_1_ = 0.57 V) implies significant overpotentials or kinetic barriers for the V(IV)/V(V) redox couple. Conversely, the smaller separation between the oxidation peak and the second reduction peak (ΔEp_2_ = 0.18 V) indicates a more favorable redox process, involving the reduction of V(III) to V(II) [35]. The CV profile for the ZnO-Fe_2_O_3_ nanocomposite electrode displays an oxidation peak at 0.2 and 0.53 V and reduction peaks at 0.37 and 0.08 V (Figure 6d). The inclusion of both ZnO and Fe_2_O_3_ in the nanocomposite electrode augments the catalytic activity, resulting in the oxidation process occurring at a higher potential compared to when ZnO or Fe_2_O_3_ is utilized alone. The first oxidation peak (0.2 V) indicates the V(II) to V(III) transition, and the second peak at (0.53 V) corresponds to the oxidation of V(IV) to V(V). The reduction peak at 0.37 V corresponds to the reduction of V(V) back to V(IV), while the second reduction peak at 0.08 V suggests an additional reduction step, possibly involving the reduction of V(III) to V(II). The separations ΔEp_1_ = 0.16 V and ΔEp_2_ = 0.45 V suggest that the redox couple involved in the reaction exhibits high irreversibility [36]. The synergistic effect of combining ZnO and Fe_2_O_3_ provides additional active sites, enhances electron transfer kinetics, and improves the surface properties of the electrode. The surface interactions between the vanadium species and the ZnO-Fe_2_O_3_ electrode influence the redox behavior, with the nanocomposite’s morphology and active sites affecting the adsorption and desorption of vanadium ions, leading to distinct redox peaks. Thus, the vanadium redox couples provide multiple sequential steps, given below [37].

The positive half-cell reaction is

(1) $${VO}^{2+}+H_{2}O↔\;{{VO}_{2}}^{+}+2H^{+}+e^{-}$$

The negative half-cell reaction is

V^3+^ + e^−^ → V^2+^ (2)

The overall oxidation/reduction reaction is

VO_2_^+^ + 2H^+^ + V^2+^ ↔ VO^2+^ + V^3+^ + H_2_O (3)

**Figure 7:**
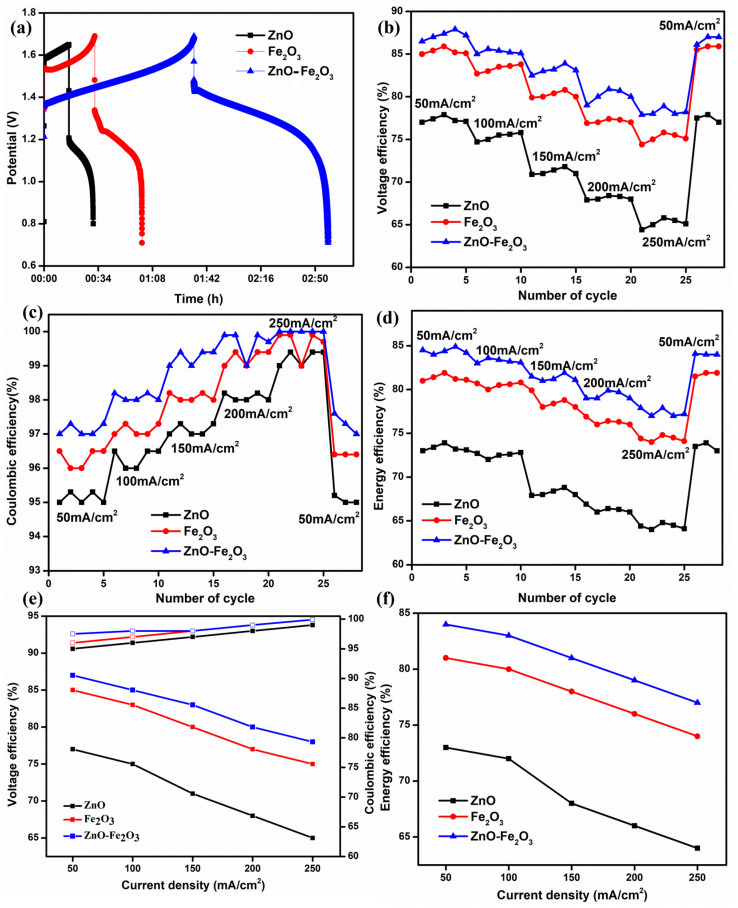
(**a**) First cycle for 3 cases at 50 mA/cm^2^, (**b**) VE, (**c**) CE, (**d**) EE, (**e**) VE, EE at different current densities, and (**f**) EE at different current densities.

The modified graphite felts were evaluated in a VRFB configuration without a flow field, incorporating a Nafion 212 proton exchange membrane and a solution of 1.6 M VOSO_4_ and 4.3 M H_2_SO_4_ serving as both the anolyte and catholyte. Figure 7a illustrates the initial charge/discharge cycle of the VRFB, which utilized ZnO, Fe_2_O_3_, and ZnO-Fe_2_O_3_ nanocomposite-modified graphite felt (GF) as the positive electrode and H GF as the negative electrode, at a current density of 50 mA/cm^2^. All three materials exhibit a low initial charging voltage and a higher discharging voltage, resulting in increased capacity. Notably, the cell with the nanocomposite electrode (ZnO-Fe_2_O_3_) demonstrated a significantly lower overpotential compared to the individual electrodes (ZnO, Fe_2_O_3_) [38]. The enhanced surface area of the nanocomposite electrode provides several benefits, including an increased number of active sites, improved mass transport, and diminished polarization (both ohmic losses and kinetic activation). As a result, the VE of the cell featuring the nanocomposite electrode was markedly higher than that of the cells with individual electrodes, culminating in superior EE [39]. The nanocomposite-modified GF exhibits VE, coulombic efficiency (CE), and EE values of 87.1%, 97.5%, and 84.9%, respectively, at a current density of 50 mA/cm^2^. The EE of the nanocomposite-modified GF is 4.1% higher than that of individual Fe_2_O_3_ and 11.2% higher than that of individual ZnO. Furthermore, the VE is 2.1% higher than that of Fe_2_O_3_ and 10.5% higher than that of ZnO. The VE, CE, and EE for all three samples at different current densities (50, 100, 150, 200, and 250 mA/cm^2^) are displayed in Figure 7b–d. According to electrochemical theory, VE and EE decrease with increasing current density due to the ohmic drop occurring in the cell. Correspondingly, CE exhibits slight variations for all samples at different current densities, as CE predominantly depends on the membrane (Nafion 212) [40]. Consequently, the cell behavior of the electrodes exhibits noticeable variances with increasing current density. All three samples begin good charging and discharging at a low current density (50 mA/cm^2^) because polarization primarily appears at high current densities. After every five cycles, the current density was increased in multiples of five, reaching up to 250 mA/cm^2^. Finally, when the current density was reverted to 50 mA/cm^2^, the VE and EE returned to their initial values, indicating that all the electrodes remained intact on the GF surface, as evidenced by the FE-SEM images. The average efficiency metrics for the various electrodes at different current densities are shown in Figure 7e,f. The results demonstrate that kinetic and ohmic losses are reduced at lower current densities. The obtained values surpass those reported in previous cells [41]. Subsequently, the modified electrodes enhance electrocatalytic activity and provide additional pathways for redox-active sites on the cathode.

**Figure 8:**
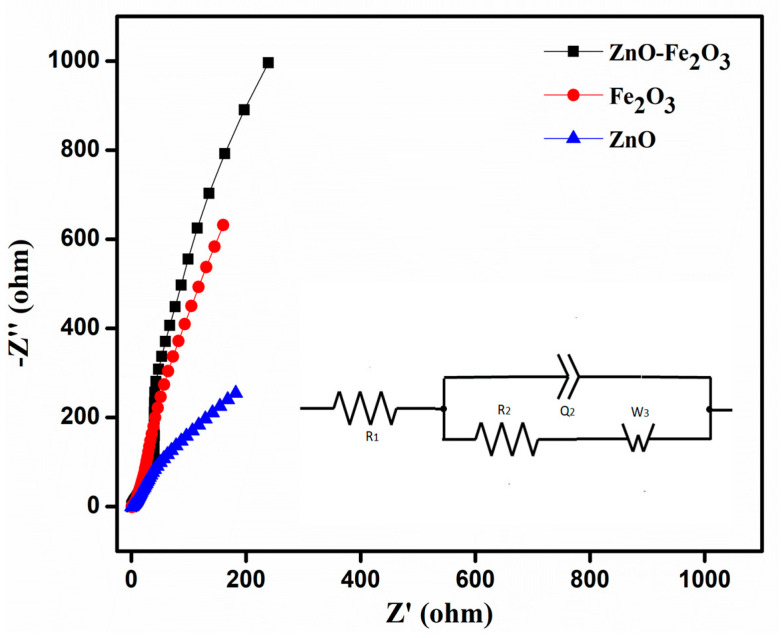
Electrochemical impedance spectroscopy (EIS) ZnO, Fe_2_O_3_, and ZnO-Fe_2_O_3_ composite, at a frequency range of 10 kHz–10 mHz.

EIS analysis was performed in the frequency range of 10 kHz–10 mHz, to calculate the solution resistance (R1), charge transfer resistance (R2), impedance, and conductivity of the as-prepared working electrode using the Nyquist plot (Figure 8). The plot shows a semicircle in the high frequency region, which corresponds to the charge transfer resistance (R2), while the initial point in the semicircle corresponds to the solution resistance (R1) contributed by the electrolyte. From the Nyquist plot, the determined R1 and R2 values of ZnO were 1.08 and 2.21 Ω, those for Fe_2_O_3_ were 1.40 and 3.17 Ω, and those for the ZnO-Fe_2_O_3_ nanocomposite were 0.81 and 1.58 Ω, respectively. From the analysis above, it is found that the combined electrochemistry of the individual pristine materials would lower the internal resistances of the ZnO-Fe_2_O_3_ nanocomposite, thereby increasing the specific capacity [42].

**Figure 9:**
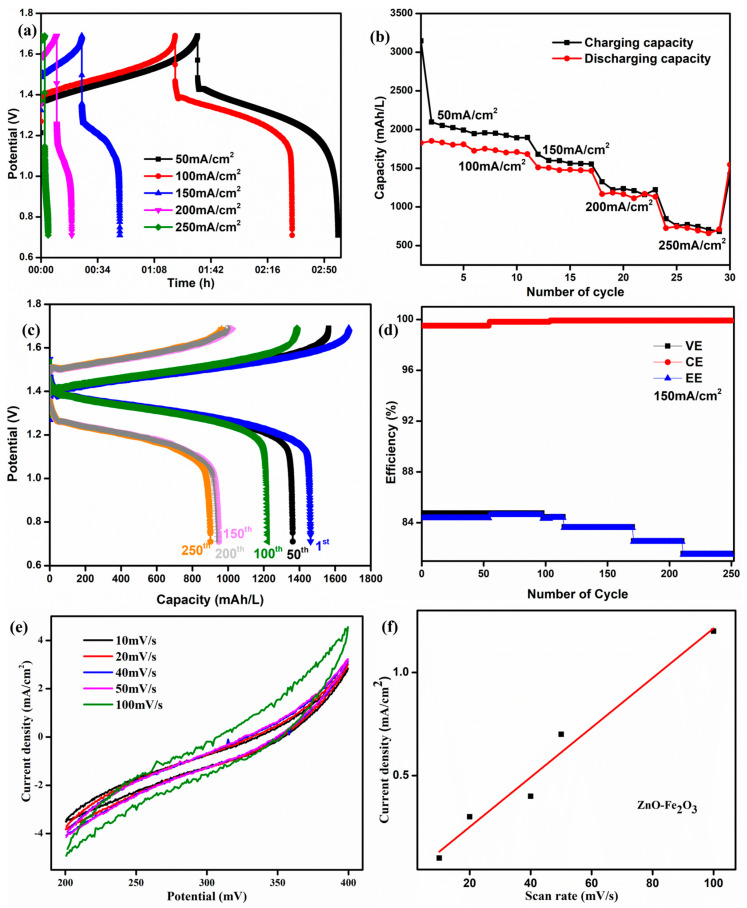
(**a**) The first cycle for the ZnO-Fe_2_O_3_ nanocomposite at different current densities. (**b**) Capacity for different current densities. (**c**) Charge/discharge capacity curve. (**d**) Efficiency for 250 cycles at 150 mA/cm^2^. (**e**) ECSA curve for the ZnO-Fe_2_O_3_ nanocomposite. (**f**) Linear fit from the ECSA.

The initial cycles of the ZnO-Fe_2_O_3_ composite at different current densities are shown in Figure 9a. At lower current densities, the longer discharge time confirms that the vanadium redox couples have smaller polarization. The corresponding specific capacity is shown in Figure 9b. At 50 mA/cm^2^, we obtained 1800 mAh/L, which is much higher than the values reported in the literature [43]. The specific capacity of the VRFB using the ZnO-Fe_2_O_3_ composite was 1489 mAh/L at 150 mA/cm^2^ for the first cycle, and it was run for 250 cycles obtain 925 mAh/L (Figure 9c). The plot of efficiency vs. cycle number at 150 mA/cm^2^ is illustrated in Figure 9d. The energy efficiency was maintained at 82% after 250 cycles. The observed energy efficiency of the cell is in good agreement with the commercial VRFB systems. The efficiency for the composite was fine-tuned within the system, exhibiting long-term cycling stability in the flow cell.

The electrochemical surface area (ECSA) of the ZnO–Fe_2_O_3_ composite was evaluated through CV, where the current density increased with higher scan rates, indicating the significant electrochemically active surface (Figure 9e). The linear relationship between peak current density and scan rate confirms a surface-controlled redox process, indicating efficient ion transfer kinetics (Figure 9f). Compared to pure Fe_2_O_3_ and the ZnO [44] materials, the composite exhibits enhanced redox activity due to the synergistic effect of Fe_2_O_3_ (high redox capability) and ZnO (improved conductivity). The ECSA was estimated using the equation [45]

ECSA = C_dl_/C_s_

where C_s_ is the specific capacitance, and C_dl_ represents the double-layer capacitance. The ZnO–Fe_2_O_3_ composite exhibited an ECSA of 14.276 cm^2^, confirming the high active surface area due to the high redox activity and conductivity of the nanocomposite-modified GF. The high ECSA and redox active sites make ZnO-Fe_2_O_3_ a promising electrode material to be employed in VRFBs.

**Figure 10:**
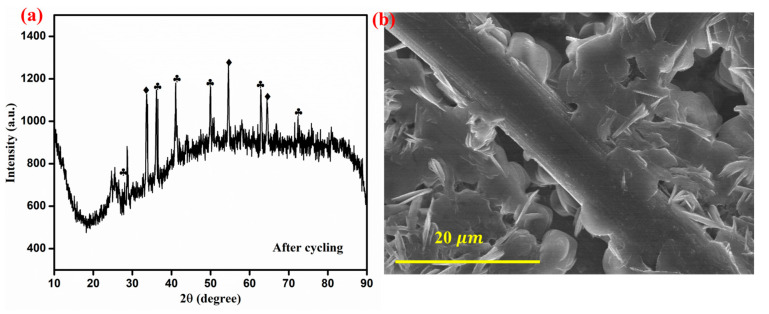
(**a**) XRD spectra after cycling (ZnO-marked by diamonds) (Fe_2_O_3_-marked by clubs), (**b**) FE-SEM image of the ZnO-Fe_2_O_3_ nanocomposite after cycling.

After 250 cycles of VRFB operation, the ZnO-Fe_2_O_3_ nanocomposite material was disassembled from the C-flow cell and then subjected to XRD and FE-SEM analyses for determining the structural and morphological changes. The XRD pattern shows multiple sharp peaks, indicating the formation of crystalline phases, possibly due to structural reorganization and vanadium-based species deposition. Additionally, the broad background signals suggest partial amorphization of the GF as a result of cycling (Figure 10a). The FE-SEM image reveals noticeable morphological changes, where nanostructures appear to have grown on the electrode surface, likely due to vanadium ion deposition. Despite these changes, the fibrous framework remains intact, revealing structural durability. Even though some degradations are evident in the FE-SEM images, the material retains its integrity, making it suitable for long-term VRFB applications [46].

## 4. Conclusions

The thermally treated graphite felt, modified with a ZnO-Fe_2_O_3_ nanocomposite, exhibited remarkable performance as a cathode material in vanadium redox flow batteries (VRFBs). This nanocomposite demonstrated significant electrocatalytic activity towards the VO^2+^/VO_2_^+^ redox couple, leading to a substantial improvement in efficiency. The findings underscore that adorning the graphite felt with the nanocomposite material generates stable active sites that effectively catalyze these electrochemical reactions. This progress represents a crucial step towards developing VRFB electrode materials with superior energy efficiency (EE). The modified cell achieved a noteworthy EE of 84% at a current density of 50 mA/cm^2^, exceeding the performance of the individual electrode materials (ZnO, Fe_2_O_3_). Additionally, this work demonstrates the nanocomposite material’s outstanding stability, sustaining a high-capacity retention rate and exhibiting no decline in EE over 250 cycles at a high current density of 250 mA/cm^2^. This study underscores an advanced modification approach for VRFB electrodes and suggests that the advancement of nanocomposite materials offers considerable potential for commercial utilization.

## Figures and Tables

**Table 1:** Values obtained from the isotherm and the pore distribution curves.

Material	SSA (m^2^/g)	Pore Volume (cm^3^/g)	Pore Diameter (nm)
ZnO	9.6	0.004872	11.9492
Fe_2_O_3_	21.3	0.008875	10.5144
ZnO-Fe_2_O_3_	23.2	0.013044	16.2271

## Data Availability

No new data were created or analyzed in this study.
